# Supplementary material for: The Systems Biology Research Tool: evolvable open-source software
Source: BMC Syst Biol. 2008 Jun 29;2:55. doi: 10.1186/1752-0509-2-55 (PMC2446383; doi:10.1186/1752-0509-2-55)
Supplement: Additional file 1 — SBRT Archive. An archive of the current version of the Systems Biology Research Tool. [file 1752-0509-2-55-S1.zip › sbrt-1.4.0/doc/developers_guide/api/sbrt/kernel/io/class-use/ResultsManager.html]

Uses of Interface sbrt.kernel.io.ResultsManager


|  |  |  |
| --- | --- | --- |
| **Overview** \| **Package** \| **Class** \| **Use** \| **Tree** \| **Deprecated** \| **Index** \| **Help** | |  |
| PREV   NEXT | **FRAMES**    **NO FRAMES**     **All Classes** |  |


---


## **Uses of Interface sbrt.kernel.io.ResultsManager**

| Packages that use ResultsManager | |
| --- | --- |
| **sbrt.kernel** | Provides interfaces to define processes. |
| **sbrt.kernel.fba.proc** | Provides classes and interfaces for executing Flux Balance Analysis processes. |
| **sbrt.kernel.io** | Provides classes and interfaces used to perform kernel-level I/O. |
| **sbrt.kernel.math.algebra.proc** | Provides classes and interfaces for executing algebra-related processes. |
| **sbrt.kernel.math.comb.proc** | Provides classes and interfaces for performing combinatorial analysis-related processes. |
| **sbrt.kernel.math.geom.proc** | Provides classes and interfaces for performing geometry-related processes. |
| **sbrt.kernel.math.graph\_theory.proc** | Provides classes and interfaces for performing graph theoretical processes. |
| **sbrt.kernel.math.stat.proc** | Provides classes and interfaces for performing statistical processes. |
| **sbrt.kernel.utilities.proc** | Provides classes and interfaces for performing utility processes. |
| **sbrt.shell.io** | Provides classes and interfaces for reading and writing files. |
| **sbrt.shell.io.fba** | Provides classes and interfaces for reading and writing files relevant to Flux Balance Analysis. |
| **sbrt.shell.mng** | Provides classes and interfaces for creating process managers. |
| **sbrt.shell.mng.fba** | Provides classes and interfaces for creating Flux Balance Analysis process managers. |

Uses of ResultsManager in sbrt.kernel

| Methods in sbrt.kernel with parameters of type ResultsManager | |
| --- | --- |
| `void` | `KernelProcess.run(ResultsManager<T> resultsManager)`             Runs this process and adds the generated results to the provided results manager via its `addData(Object)` method. |

Uses of ResultsManager in sbrt.kernel.fba.proc

| Methods in sbrt.kernel.fba.proc with parameters of type ResultsManager | |
| --- | --- |
| `void` | `CdHarFluxVectorProcess.run(ResultsManager<CdHarFluxVectorComputer.Results> resultsManager)`             Generates the specified number of random flux vectors and adds each to the provided results manager. |
| `void` | `SvCdHarFluxVectorProcess.run(ResultsManager<CdHarFluxVectorComputer.Results> resultsManager)`             Runs this process and adds the generated results to the provided results manager via its `addData(Object)` method. |
| `void` | `CycleIdProcess.run(ResultsManager<DoubleMatrix<?,java.lang.String>> resultsManager)`             Runs this process and adds the generated results to the provided results manager via its `addData(Object)` method. |
| `void` | `ExtremeCurrentProcess.run(ResultsManager<DoubleMatrix<?,java.lang.String>> resultsManager)`             Runs this process and adds the generated results to the provided results manager via its `addData(Object)` method. |
| `void` | `CvFbaOptimizationExperiment.run(ResultsManager<FbaOptComputer.Results> resultsManager)`             Runs this process and adds the generated results to the provided results manager via its `addData(Object)` method. |
| `void` | `ObjectiveFunctionAnalysis.run(ResultsManager<FbaOptComputer.Results> resultsManager)`             Runs this process and adds the generated results to the provided results manager via its `addData(Object)` method. |
| `void` | `CatalystDeletionProcess.run(ResultsManager<FbaOptComputer.Results> resultsManager)`             Runs this process and adds the generated results to the provided results manager via its `addData(Object)` method. |
| `void` | `RxnDeletionProcess.run(ResultsManager<FbaOptComputer.Results> resultsManager)`             Runs this process and adds the generated results to the provided results manager via its `addData(Object)` method. |
| `void` | `CvRxnDeletionProcess.run(ResultsManager<FbaOptComputer.Results> resultsManager)`             Runs this process and adds the generated results to the provided results manager via its `addData(Object)` method. |
| `void` | `StoichVariationProcess.run(ResultsManager<FbaOptComputer.Results> resultsManager)`             Runs this process and adds the generated results to the provided results manager via its `addData(Object)` method. |
| `void` | `CvObjectiveFunctionProcess.run(ResultsManager<FbaOptComputer.Results> resultsManager)`             Runs this process and adds the generated results to the provided results manager via its `addData(Object)` method. |
| `void` | `FbaOptimizationProcess.run(ResultsManager<FbaOptComputer.Results> resultsManager)`             Computes the optimal value of the current objective function and adds the results to the provided results manager via its `addData(Object)` method. |
| `void` | `CvCatalystDeletionProcess.run(ResultsManager<FbaOptComputer.Results> resultsManager)`             Runs this process and adds the generated results to the provided results manager via its `addData(Object)` method. |
| `void` | `FluxActivityProcess.run(ResultsManager<FluxActivityProcess.Results> resultsManager)`             Iterates thru the flux vector source set via `setFluxVectorSource(Iterable)`, counts the number of active and inactive fluxes in each vector, and adds the results of each to the provided results manager. |
| `void` | `FluxPlasticityProcess.run(ResultsManager<FluxPlasticityProcess.Results> resultsManager)`             Iterates thru the flux range source set via `setFluxRangeSource(Iterable)`, computes the flux plasticity measures, and adds the results of each to the provided results manager. |
| `void` | `InitialPointGenerator.run(ResultsManager<InitialPointComputer.Results> resultsManager)`             Generates an initial point and adds it to the provided results manager. |
| `void` | `SvInitialPointProcess.run(ResultsManager<InitialPointComputer.Results> resultsManager)`             Runs this process and adds the generated results to the provided results manager via its `addData(Object)` method. |
| `void` | `RandomObjectiveGenerator.run(ResultsManager<LinearComb<java.lang.String>> resultsManager)`             Generates random objective functions and adds them to the provided results manager. |
| `void` | `FbaSolverProcess.run(ResultsManager<LinearSystemSolution<java.lang.String>> resultsManager)`             Solves Sv = 0 and adds the result to the provided manager. |
| `void` | `FluxIntervalProcess.run(ResultsManager<java.util.Map<java.lang.String,Interval>> resultsManager)`             Computes the flux ranges of the reactions with the current names and adds them to the provided results manager. |
| `void` | `RandomEnvGeneratorV1.run(ResultsManager<java.util.Map<java.lang.String,Interval>> resultsManager)`             Generates a random environment and adds it to the provided results manager. |
| `void` | `CvFluxIntervalProcess.run(ResultsManager<java.util.Map<java.lang.String,Interval>> resultsManager)`             Runs this process and adds the generated results to the provided results manager via its `addData(Object)` method. |
| `void` | `RandomConstraintsGeneratorV1.run(ResultsManager<java.util.Map<java.lang.String,Interval>> resultsManager)`             Generates sets of random flux constraints and adds them to the provided results manager. |
| `void` | `FluxCapProcess.run(ResultsManager<java.util.Map<java.lang.String,java.util.Set<LinearComb<java.lang.String>>>> resultsManager)`             Computes the flux caps and adds the result to the provided manager. |
| `void` | `NetworkInfoProcess.run(ResultsManager<NetworkInfoProcess.Results> resultsManager)`             Adds the information gathered about the fluxome to the provided results manager. |
| `void` | `FluxomeReducerProcess.run(ResultsManager<T> resultsManager)`             Runs this process and adds the generated results to the provided results manager via its `addData(Object)` method. |

Uses of ResultsManager in sbrt.kernel.io

| Classes in sbrt.kernel.io that implement ResultsManager | |
| --- | --- |
| `class` | `AbstractResultsManager<R>`             This class is a skeleton implemenation of `ResultsManager`. |
| `class` | `ListResultsManager<R>`             This class is used to store a list of results that can be easily retrieved. |
| `class` | `SingleRm<R>`             This class is used to store a single result that can be easily retrieved. |

Uses of ResultsManager in sbrt.kernel.math.algebra.proc

| Methods in sbrt.kernel.math.algebra.proc with parameters of type ResultsManager | |
| --- | --- |
| `void` | `LinearSolverProcess.run(ResultsManager<LinearSystemSolution<V>> resultsManager)`             Solves the system of linear equations and adds the solution to the provided results manager. |
| `void` | `VectorIntersection.run(ResultsManager<java.util.Map<K,V>> resultsManager)`             and adds the results to the provided results manager. |

Uses of ResultsManager in sbrt.kernel.math.comb.proc

| Methods in sbrt.kernel.math.comb.proc with parameters of type ResultsManager | |
| --- | --- |
| `void` | `SingleElementUnionProc.run(ResultsManager<java.util.Set<java.util.Set<E>>> resultsManager)`             Computes the single-element unions of the provided collection of sets, and adds the results to the provided results manager. |
| `void` | `StrictSingleElementUnionProc.run(ResultsManager<java.util.Set<java.util.Set<E>>> resultsManager)`             Computes the strict single-element unions of the provided collection of sets, and adds the results to the provided results manager. |

Uses of ResultsManager in sbrt.kernel.math.geom.proc

| Methods in sbrt.kernel.math.geom.proc with parameters of type ResultsManager | |
| --- | --- |
| `void` | `CdHarProcess.run(ResultsManager<CdHarComputer.Results> resultsManager)`             Adds random points to the provided results manager. |

Uses of ResultsManager in sbrt.kernel.math.graph\_theory.proc

| Methods in sbrt.kernel.math.graph\_theory.proc with parameters of type ResultsManager | |
| --- | --- |
| `void` | `CycleIdProcess.run(ResultsManager<Path<N>> resultsManager)`             Adds to the provided results manager each identified cycle in the current graph. |
| `void` | `PathIdProcess.run(ResultsManager<Path<N>> resultsManager)`             Adds to the provided results manager each computed path in the current graph. |

Uses of ResultsManager in sbrt.kernel.math.stat.proc

| Methods in sbrt.kernel.math.stat.proc with parameters of type ResultsManager | |
| --- | --- |
| `void` | `MwUAnalysis.run(ResultsManager<MannWhitneyResults> resultsManager)`             Computes the Mann-Whitney U statistic of the samples A and B, and adds the results to the provided results manager. |
| `void` | `CorrelationAnalysis.run(ResultsManager<T> resultsManager)`             Computes the correlation statistic between the x and y values, and adds the results to the provided results manager. |
| `void` | `MultipleCorrelationAnalysis.run(ResultsManager<T> resultsManager)`             Computes the correlation statistic between the x and y values, and adds the results to the provided results manager. |

Uses of ResultsManager in sbrt.kernel.utilities.proc

| Methods in sbrt.kernel.utilities.proc with parameters of type ResultsManager | |
| --- | --- |
| `void` | `VectorComparison.run(ResultsManager<java.util.Map<V,java.lang.Double>> resultsManager)`             Adds to the provided results manager the variables (keys) whose intervals (values) are unequal within the provided tolerance. |
| `void` | `VariableParticipation.run(ResultsManager<java.util.Map<V,java.util.List<E>>> resultsManager)`             Adds to the provided results manager a map of variables mapped to a list of mathematical expressions in which they participate. |
| `void` | `IntervalComparison.run(ResultsManager<java.util.Set<IntervalComparer.Diff<V>>> resultsManager)`             Adds to the provided results manager the variables (keys) whose intervals (values) are unequal within the provided tolerance. |

Uses of ResultsManager in sbrt.shell.io

| Subinterfaces of ResultsManager in sbrt.shell.io | |
| --- | --- |
| `interface` | `MapOutputFile<K,V>`             This interface is used to represent output files that maps can be written to. |
| `interface` | `MapTextOutputFile<K,V>`             This interface is used to represent output files that maps can be written to as text. |
| `interface` | `OutputFile<T>`             This interface is used to represent output files. |

| Classes in sbrt.shell.io that implement ResultsManager | |
| --- | --- |
| `class` | `AbstractMapOutputFile<K,V>`             This abstract class is a skeleton implementation of `MapTextOutputFile`. |
| `class` | `ComoFile<K,V>`             This class is used to write maps to text files in a column-oriented way. |
| `class` | `MultiOutputFiles<F extends OutputFile<R>,R>`             This class is used to create multiple output files. |
| `class` | `RomoFile<K,V>`             This class is used to write maps to text files in a row-oriented way. |
| `class` | `SerializedOutputFile<E>`             This class is used to write serialized objects to output files. |
| `class` | `SmoFile<K,V>`             This class is used to write serialized maps to output files. |
| `class` | `TextOutputFile<T>`             This class is used to represent output files containing plain text. |

Uses of ResultsManager in sbrt.shell.io.fba

| Subinterfaces of ResultsManager in sbrt.shell.io.fba | |
| --- | --- |
| `interface` | `FbaOptOutputFile`             This interface is used to represent FBA optimization output files. |

| Classes in sbrt.shell.io.fba that implement ResultsManager | |
| --- | --- |
| `class` | `ColumnOrFbaOptOutputFile`             This class is used to write column-oriented output files containing FBA optimization data. |
| `class` | `RowOrFbaOptOutputFile`             This class is used to write row-oriented output files containing FBA optimization data. |
| `class` | `SerFbaOptOutputFile`             This class is used to write output files containing FBA optimization data as serialized maps. |

Uses of ResultsManager in sbrt.shell.mng

| Methods in sbrt.shell.mng with parameters of type ResultsManager | |
| --- | --- |
| `static void` | `AbstractProcessManager.closeRm(ResultsManager rm, java.io.PrintWriter out)`             Closes the provided file and prints a descriptive message to the provided print writer. |
| `static <T> void` | `AbstractProcessManager.runProcess(MonitoredProcess<T> process, ResultsManager<T> resultsManager, java.io.PrintWriter out)`             Runs the provided process using the provided results manager and prints descriptive progress messages to the provided print writer. |

Uses of ResultsManager in sbrt.shell.mng.fba

| Classes in sbrt.shell.mng.fba that implement ResultsManager | |
| --- | --- |
| `protected static class` | `ExtremeCurrentManager.Rm` |

---


|  |  |  |
| --- | --- | --- |
| **Overview** \| **Package** \| **Class** \| **Use** \| **Tree** \| **Deprecated** \| **Index** \| **Help** | |  |
| PREV   NEXT | **FRAMES**    **NO FRAMES**     **All Classes** |  |


---
